# Racial and Gender-Based Differences in COVID-19

**DOI:** 10.3389/fpubh.2020.00418

**Published:** 2020-07-28

**Authors:** Jonathan Kopel, Abhilash Perisetti, Ali Roghani, Muhammad Aziz, Mahesh Gajendran, Hemant Goyal

**Affiliations:** ^1^Texas Tech University Health Sciences Center, Lubbock, TX, United States; ^2^Department of Gastroenterology and Hepatology, University of Arkansas for Medical Sciences, Little Rock, AR, United States; ^3^Department of Internal Medicine, The University of Toledo, Toledo, OH, United States; ^4^Paul L. Foster School of Medicine, Texas Tech University Health Sciences Center El Paso, El Paso, TX, United States; ^5^The Wright Center for Graduate Medical Education, Scranton, PA, United States

**Keywords:** coronavirus, COVID-19, SARS-CoV-2, pandemic, sex, gender, race

## Abstract

The novel coronavirus disease (COVID-19) has become a global health crisis since its first appearance in Wuhan, China. Current epidemiological studies suggest that COVID-19 affects older patients with multiple comorbidities, such as hypertension, obesity, and chronic lung diseases. The differences in the incidence and severity of COVID-19 are likely to be multifaceted, depending on various biological, social, and economical factors. Specifically, the socioeconomic differences and psychological impact of COVID-19 affecting males and females are essential in pandemic mitigation and preparedness. Previous clinical studies have shown that females are less susceptible to acquire viral infections and reduced cytokine production. Female patients have a higher macrophage and neutrophil activity as well as antibody production and response. Furthermore, *in-vivo* studies of the angiotensin-converting enzyme 2 (ACE2) showed higher expression in the kidneys of male than female patients, which may explain the differences in susceptibility and progression of COVID-19 between male and female patients. However, it remains unknown whether the expression of ACE2 differs in the lungs of male or female patients. Disparities in healthcare access and socioeconomic status between ethnic groups may influence COVID-19 rates. Ethnic groups often have higher levels of medical comorbidities and lower socioeconomic status, which may increase their risk of contracting COVID-19 through weak cell-mediated immunity. In this article, we examine the current literature on the gender and racial differences among COVID-19 patients and further examine the possible biological mechanisms underlying these differences.

## Introduction

The novel coronavirus disease (COVID-19) is caused by severe acute respiratory syndrome coronavirus-2 (SARS-CoV-2). The first reported case of COVID-19 was in December 2019 in Wuhan, China. The disease has continued to spread globally and was classified as a pandemic in March by the World Health Organization. Coronaviruses belong to a family of single-stranded RNA viruses, which cause several respiratory, gastrointestinal, hepatic, and neurologic diseases (1–3). Similar to the viruses causing Severe Acute Respiratory Syndrome (SARS) and Middle East Respiratory Syndrome (MERS), the SARS-CoV-2 is the seventh coronavirus (CoV) identified that can infect humans (2, 4, 5). Patients infected with SARS-CoV-2 have an incubation period of 3–14 days with a mean period of 5 days (1, 5, 6). The most common symptoms of COVID-19 include fever, cough, fatigue, and bleeding (2, 4, 5, 7–11). Other symptoms include taste changes, headache, abdominal pain, diarrhea, and gastrointestinal bleeding (2, 4, 5, 7–11). If left untreated, COVID-19 can lead to pneumonia, acute respiratory distress syndrome (ARDS), and acute respiratory failure resulting in death (1, 5, 6). The number one risk factor for severe disease is age with the severity increasing with the presence of comorbidities, such as heart and lung diseases (1, 5, 6). However, the effects of gender and ethnicity on SARS-CoV-2 infection rates and severity remain an area of active investigation.

The original clinical reports from China suggested that the COVID-19 virus infected both men and women equally; further studies suggested that sex differences exist in both mortality and infection susceptibility for SARS-CoV-2 (12, 13). From a socioeconomic perspective, school closures force more women with families to provide informal care for their immediate families, which limits women's work and economic opportunities for advancement (13). These differences are further compounded by the unique physical, sanitary, and security needs of women in quarantine conditions compared to men (13). Furthermore, data from the Centers for Disease Control (CDC) suggest that ethnic differences between COVID-19 patients may influence susceptibility and mortality. However, the mechanism for such differences remains mostly unknown (14). Another theory for these differences is related to differences in the expression of angiotensin-converting enzyme 2 (ACE2) receptors, which is the primary receptor for viral entry into the cells. In this article, we aim to elaborate on the effect of gender and ethnic backgrounds on COVID-19 infection and related mortality.

## Gender and COVID-19

### Studies With Male Predominance

The first cases of COVID-19 that occurred in China indicated the presence of gender differences (13). Initial reports estimated that 60% of COVID-19 patients were male (13). A study examining 799 patients in the Tongji Hospital in Wuhan, China found that of 113 COVID-19 deaths, 27% were female, and 73% were male (15). The authors concluded that the fatality rate was higher in men, possibly due to an increased prevalence of cardiopulmonary disease and smoking (15). Men were also more likely to develop heightened systemic inflammation, multi-organ dysfunction, and cardiac injury (15). A similar study of 54 deceased COVID-19 patients in South Korea showed that 61% of the patients were male.

A subsequent study examining 155 consecutive patients with confirmed COVID-19 in Zhongnan Hospital of Wuhan University found that 56% of the patients were male (16). A multivariate analysis performed in this study showed that male sex was a significant risk factor (OR: 2.206, 95% CI: 1.012–4.809) for prolonged (>14 days) COVID-19 symptoms (16). However, since 49% of these patients had chronic diseases, the authors believed that older male patients might have an increased risk for COVID-19, resulting in longer hospital stays and slow recovery (16). Similarly, another study showed that the male gender was a significant factor for COVID-19 infection on logistic regression analysis, with elderly male patients being at higher risk for the virus (17). Another study examined 46 deceased COVID-19 patients and found that men accounted for 67% of the fatalities (18). However, higher mortality in males could be a reflection of increased risk and prevalence of COVID-19 among male patients rather than being correlated with the male gender (18). A study examining 133 COVID-19 patients in Wuhan China, reported a similar male-predominance (58% male vs. 42% female) of COVID-19 infections (19). The study found that male patients were more likely than females (odds ratio: 3.24; 95% CI: 1.31–8.02) to shed the COVID-19 (20). Specifically, male patients continued to shed the COVID-19 virus for 18 days, while females shed the virus for 15.2 days (19).

Given the potential gender disparity, a recent study examined 4,880 COVID-19 patients who either had respiratory symptoms or close contact with a COVID-19 patients in Wuhan, China using quantitative RT-PCR (qRT-PCR) from nasopharyngeal samples. The study found no significant gender differences in the sample, which included 2,251 (46%) male and 2,629 (54%) female patients (17). However, 36.71% of females and 40.43% of males tested positive for COVID-19; furthermore, the positive rate of COVID-19 diagnosis using qRT-PCR was higher in males than females (17). Similarly, the positive rate for COVID-19 diagnosis also increased from 24.9 to 61.81% between younger and older patients (17). A recent study from COVID-19 patients in China showed that both the severity and mortality rates were worse among men than women (21). Specifically, men were over two times more likely to die from COVID-19 than women (21).

### Studies With Female Predominance

Although the first reports in China showed a predominance of male COVID-19 patients, recent studies suggest that females may be at higher risk for COVID-19. The Korean Society of Infectious Diseases collected data on 4,212 COVID-19 patients, which showed that 37.7% were males while 62.3% were female (22). These results are in contrast to Chinese data, which estimated ~51% of COVID-19 patients were male (22). The authors suggest the difference may reflect differences in social activities from different countries; in South Korea, the largest social age group are in their 20s. Furthermore, contact tracing of the COVID-19 outbreak in South Korea suggested that female practitioners of the Daegu religious sect may have contributed to the COVID-19 outbreak. Therefore, gender disparities in COVID-19 may reflect social and cultural differences between different countries (22). In a similar study in Qingdao City, China, examining 44 COVID-19 patients showed that 66% were female. The female predominance reported in this study was likely due to the small sample size during the early stages of the COVID-19 epidemic (23).

A larger study from the Zhejiang Province of China examined the gender distribution of COVID-19 patients in young and elderly patients (24). Young patient populations showed no significant difference in gender distribution (54% male 46% female). In contrast, the elderly (>60 years) COVID-19 patients were predominately female (43% male vs. 57% female) (24). The differences in gender distribution are likely from increased medical comorbidities reported among elderly patients (older vs. younger groups: 55.15 vs. 21.93%) (24). Lastly, a multicenter European study examining 417 COVID-19 patients showed a higher proportion of COVID-19 patients were female (63%) than male (37%) (25). Interestingly, the study also found that female COVID-19 patients were more likely than males to be affected by olfactory and gustatory dysfunctions (25). It is unknown what biological process may be involved in female patients exhibiting a proportion of sensory dysfunction related to COVID-19 (25).

### Studies With No Gender Predominance

Despite some studies showing gender differences in the incidence and case fatality rate in COVID-19 patients, a growing number of studies show no gender differences in SARS-CoV-2 infections. A study in Jiangsu Province, China, examined 80 patients with COVID-19 who found that men (49%) and women (51%) were equally affected (26). The authors of the study noted that the lack of gender differences could be related to the small sample size or the mode of transmission during the early stages of the pandemic (26). A similar study involving 135 patients, an equal distribution between men (53%) and women (47%) were noted with an average age of 47 years (27).

A study involving patients on a Japanese cruise ship found that among the 634 people who tested positive for COVID-19, 49% of cases were female and 51% male (20). The cases were from a total of 28 countries, including Japan (270 cases), the United States (88 cases), China (58 cases; including 30 from Hong Kong), Philippines (54 cases), Canada (51 cases), and Australia (49 cases) (20). Given the assortment of different ethnic groups in close proximity, this study suggests that COVID-19 infection rates may not depend on gender but may be reflective of underlying health status, comorbidities, and social factors within a given population.

### Reproductive and Psychological Challenges of COVID-19 on Females

Despite the rapid spread of the disease, the COVID-19 outbreak has revealed unique challenges for both men and women. For pregnant women infected with COVID-19, there are reports of fetal distress, preterm delivery, and intrauterine virus transmission in the third trimester; however, the lack of information has produced much uncertainty concerning the overall health of the mother and fetus exposed to COVID-19 (28). Therefore, pregnant women are treated aggressively if exposed to COVID-19 and remain a vulnerable risk group to the COVID-19 virus (28). Furthermore, pregnant women face additional challenges with work, child-rearing, and maternal services, which further increase the risk of exposure to COVID-19 (28). Women may also have limited access to acute and emergency reproductive services forcing many women to travel long distances to safe medical facilities or have their child delivered at home in developing countries (28). In addition, cesarean sections and abortion care are also limited due to staff shortages and increased risk of COVID-19 infections in surgical wards (28). In poorer countries, these limitations are further compounded by the limited access to routine clinical care for contraceptive counseling or other reproductive health services (28).

As a result, the females are associated with a more significant psychological impact leading to higher levels of stress, anxiety, and depression (29, 30). A study following the outbreak in Wuhan, China, found that the level of post-traumatic stress syndrome (PTSS) was 7% in women (21.9%) who reported higher negative alterations in mood and hyper-arousal than men (14.6%) (30). A survey conducted by the Kaiser Family Foundation in the US telephone interviewed 1,126 adults to determine the differences in men and women responding to the COVID-19 pandemic (31). The survey found that more women than men worry that they or someone in their family will get sick from the coronavirus (68 vs. 56%) or are concerned about losing income due to a workplace closure or reduced hours (50 vs. 42%) (31). In addition, more women compared to men worry that they would put themselves at risk of exposure to coronavirus because they cannot afford to stay home and miss work (39 and 31%). Women also reported having more part-time jobs than men (13 vs. 9%) and worried more than men about whether they would be able to afford testing or treatment for coronavirus if they need it (40 vs. 31%) (31). Given the increased stress reported in this survey, women were also asked questions about their mental health. The survey found that more women (16%) than men (11%) felt that worry or stress related to COVID-19 would have a significantly negative impact on their mental health. Furthermore, four in 10 women (36%) and three in 10 men (27%) felt worried or stressed about how coronavirus has had some impact on their mental health (31).

Despite the emotional challenges faced by women, women reported taking more precautions to reduce their exposure and spread of COVID-19 compared to men. The survey found that more women compared to men say they decided not to travel or changed travel plans (47 vs. 37%), or reported canceling plans to attend large gatherings (43 vs. 36%). Furthermore, women were more likely than men to stock food, household supplies, or prescription medications (39 vs. 30%), and planned to stay at home instead of going to work, school, or other regular activities (30 vs. 22%). In this respect, women may act as a safety net and an essential component for maintaining social distancing with their families and society at large by balancing several responsibilities. Studies show that women play an essential role in the stages of public health management, including planning, decision-making, and emergency response systems (32). Furthermore, women are the primary caregiver for the young, the elderly, and sick in most households and healthcare facilities (32). Despite this, women remain under-represented in most political and healthcare organizations (32). Specifically, political and healthcare organizations with a higher representation of women had a lower number of COVID-19 cases and hospitalizations (32). Without a long-term policy response, including more female representation, these issues will persist long after the COVID-19 pandemic has passed.

## Race and COVID-19 Infection

Current epidemiological data on COVID-19 suggests that minority groups may also be more susceptible to COVID-19 infections (14). A study conducted by the CDC and the COVID-19–Associated Hospitalization Surveillance Network during March 2020 examined 1,482 hospitalized patients across 14 states in the US (14). The study found that 54 and 46% of hospitalized patients were male and female, respectively (14). Furthermore, the COVID-19-associated hospitalization rates were higher among males than among females (5.1 vs. 4.1 per 100,000 population) (14). Furthermore, CDC and COVID-19–Associated Hospitalization Surveillance Network examined 580 of the 1,482 COVID-19 patients with race/ethnicity data and found that 45.0% were Caucasian, 33% African-American, 8% Hispanic, 5% Asian, <1% American Indian/Alaskan Native, and 7.9% were of other or unknown race (14). These results are impressive since 33% of hospitalized patients were African American even though they constitute 13% of the United States (US) population. In contrast, 8% of hospitalized patients were Hispanics, who make up 18% of the US population, and 45% of hospitalized COVID-19 patients were Caucasian, who make 76% of the US population.

However, the study noted racial distributions of hospitalized COVID-19 varied state by state depending on the population of interest (14). Furthermore, minority populations, such as African Americans, are more likely to have diabetes, hypertension, obesity, asthma, and heart disease, which increases their risk of contracting COVID-19. The CDC and COVID-19–Associated Hospitalization Surveillance Network reported that 89% of hospitalized COVID-19 patients had some form of a pre-existing condition. Specifically, 50% of hospitalized patients had hypertension, 48% had obesity, 35% had chronic lung diseases, 28% had diabetes mellitus, and 28% had cardiovascular disease (14). A previous study examining cytomegalovirus (CMV) among different socioeconomic groups in the US found that a reduced socioeconomic status was associated with a more inferior cell-mediated immunity (33). The study suggested that patients with a lower socioeconomic status predispose them to reduced access to medical care, multiple comorbidities, poor diet, and life stressors that could weaken their immune system.

Given that many minority populations have higher proportions of patients with low socioeconomic status, this may be a contributing factor to the higher prevalence of COVID-19 infections (33–35). Current COVID-19 guidelines encourage clinicians to perform preventive measures, such as social distancing, respiratory hygiene, and wearing face coverings in public settings, to protect older adults and persons with underlying medical conditions (14). There may exist some racial disparity in COVID-19 infections, given the data published on SARS infections (36). A previous study examining polymorphisms in mannose-binding lectin (MBL) genes found that MBL gene polymorphisms were associated with increased susceptibility to SARS-CoV infection (36). Further data collection and research are needed to determine if a racial disparity exists with COVID-19 and which socioeconomic and biological factors are involved.

Although few studies have examined the biological mechanisms underlying ethnic differences of COVID-19 infection susceptibility, a recent study noted that ACE2 expression could vary among Asians (significantly higher) compared to African Americans and Caucasians (37). Though this might explore susceptibility patterns among different ethnicities, larger studies are needed to validate these. Therefore, it remains open whether gender or ethnic differences exist with the expression of ACE2, and ultimately, the pathogenesis of COVID-19.

The major limitation in determining whether gender or ethnic differences exist for COVID-19 patients is the lack of pre-clinical and clinical studies. The significant studies for documenting the epidemiological data for COVID-19 patients were from Wuhan, China. Only two studies included data outside of Wuhan, China, and these data sets were located in Asian countries (Japan and Korea). Therefore, there is a significant bias in the ethnic group represented in these samples. Besides, the limited number of patients diagnosed with COVID-19 may be much higher than reported, given the lack of systematic testing during the initial stages of the outbreak. Furthermore, patients presenting with mild symptoms of cough, fever, and headache may have been misdiagnosed with influenza rather than COVID-19. Given the rapid changes in this pandemic, the data on infection rate, morbidity, and mortality between male and female COVID-19 patients will likely improve with larger sample sizes and greater distribution of age and ethnic backgrounds.

## Putative Mechanisms For Gender Differences Among COVID-19 Patients

Although the precise mechanism of gender differences in COVID-19 remains unknown, previous studies provide insights into possible mechanisms. Previous studies have shown females have increased resistance to viral, bacterial, fungal, and parasitic organisms than males (38). Specifically, females are less susceptible to microbial infections by mounting a higher innate immune response than males (39, 40). Women produce a higher expression of the inflammatory and cytotoxic proteins, including interferon-g (IFN-g), lymphotoxin b (LTb), granzyme A (GZMA), interleukin-12 receptor b2 (IL12Rb2), and granulysin (GNLY) (39). Furthermore, female patients are less likely to produce extreme immune responses to bacterial or viral infections than male patients leading to sepsis. A study examining gender differences in sepsis patients found that male patients have higher circulating levels of TNF-α than female patients, which are correlated with a worse prognosis (41). Female sepsis patients were protected from sepsis due to the increased production of the immunosuppressive cytokine IL-10 (41). Specifically, female patients produce lower levels of inflammatory mediators and increase the production of immunosuppressive molecules to reduce systemic inflammation. The protection of females to microbial and viral affections is attributed to the protection provided by the X chromosome and sex hormones, which modulate the innate and adaptive immunity (38). In contrast, males are at higher risk of developing cancers as opposed to females who have a higher risk of autoimmune diseases (38).

### Hormonal Effect

Estrogen has been well-documented as a positive stimulator of the immune response, particularly with increasing the activity and proliferation of T-cells (42). Estrogen suppresses the immune system by reducing the production of IL-1β, IL-6, and TNF by monocytes (43). Estrogen also reduces the expression of nitric oxide synthase, which impairs chemotaxis of neutrophils (44, 45). Furthermore, estrogen increases the expression of Toll-like Receptor 4 (TLR4) and CD14 expression on macrophages and the differentiation and activation of dendritic cells (38). Lastly, estrogen increases humoral responses from B lymphocytes producing more antibodies in females than males through enhancing IgG and IgM antibodies (38).

Testosterone shows several immunosuppressive functions by reducing cytokine production and proliferation of lymphocytes (38). It increases neutrophil activation in non-infectious states while reducing the expression of TLR4 (38). It reduces IgM and IgG production directly and by reducing the production of IL-6 by monocytes (38). As a result, men with higher testosterone levels have been reported to have lower titers of antibodies after vaccination compared to women who have lower testosterone levels (38). Therefore, the levels of testosterone and estrogen between men and women could predispose individuals to different levels of severity in COVID-19 symptoms.

### Infection Susceptibility

Women are less susceptible to viral infections than men due to their mounting of more robust immune responses. A study comparing HIV-1 infections in men and women showed that untreated HIV-1 infected women had 40% less circulating viral RNA and greater activation in CD8^+^ T cells than men (46, 47). Similar studies with Hepatitis B and C viruses, HIV, Hantavirus, West Nile Virus infections, and influenza viruses showed that men were more susceptible than females to viral infections (48). However, the study also found that the hyperactive response in female immune systems to viral infections may increase symptom severity and pathological effects than observed in male patients (49). The authors hypothesized that effect could result from an increase in the production of cytokines, chemokines, and interferons in females than males (50). Following the elimination of the viral infection, females maintain elevated immune responses that can increase the risk of post-infection complications compared to men (50). Given these disparities in viral symptoms, female patients infected with COVID-19 may experience more long-term complications than men. Further investigation into the immune response to COVID-19 between male and female patients will be needed.

### Gender Differences in ACE2

It remains uncertain whether there are gender differences in the expression of ACE2 receptor, which is the protein involved in the first step of viral entry for COVID-19 (51). ACE-2 is a type-1 transmembrane metallo-carboxypeptidase that regulates blood pressure through the renin-angiotensin systems (RAS) (52). The ACE-2 degrades Angiotensin II to generate Angiotensin 1–7, thereby negatively regulating RAS (53, 54). Although ACE-2 is expressed mostly in the vascular endothelial cells, the renal tubular epithelium, and in Leydig cells in the testes, ACE-2 has also been detected in several other organs and tissues, such as the nasopharynx, lung, stomach, small intestine, colon, lymph nodes, thymus, bone marrow, spleen, liver, kidney, and brain (55, 56).

*In vivo* studies suggest that ACE2 expression in the kidney is higher in males than females due to the presence of testosterone and estrogen regulatory activities on post-transcriptional and post-translational mechanisms on ACE2 expression (57, 58). However, the same study examining rat lung ACE-2 expression also showed a decreased expression of ACE-2 with increasing rat age (59). Furthermore, there was no difference observed in the ACE2 expression in the heart and lungs of both rats and mice (60, 61). Interestingly, the expression of ACE2 changes through the life-span of female rats through fluctuations in estradiol (E_2_); in particular, the expression of ACE2 increases significantly during pregnancy (62). Together, these studies suggest a combination of social and biological differences (e.g., ACE-2 expression) in pregnant female patients may predispose them to COVID19. Medical comorbidities with ACE-2 expression may also explain the increased susceptibility of pregnant female patients to COVID-19. However, further histological and pathology studies are needed to examine the influence of age and gender on the expression of lung ACE-2 and the risk of patients for COVID-19 infection.

Currently, smaller sample sizes or cases are being reported in the literature to assess the gender and ethnic differences of COVID-19 patients (63). Furthermore, the lack of systematic testing across the world limits the accuracy of epidemiological data for the distribution of COVID-19 patients. Second, the morbidity and mortality rates for gender and ethnic groups should be stratified in future epidemiological studies on COVID-19 to assess the differences in healthcare interventions and outcomes for each group (63). Third, there needs to be an increase in scientific and pathological studies assessing the expression and activity of ACE-2 to identify which patient populations are most at risk. Currently, there have been few studies investigating ACE-2 expression in COVID-19 patients and whether ACE-2 inhibitors may provide therapeutic benefit. Further genetic and biomedical studies are needed to determine if other biomarkers exist for evaluating susceptible populations and guiding patient management. This information would provide clinicians a broader perspective of the biological, social, and economic factors influencing the susceptibility and management of COVID-19 patients.

## Gaps in Knowledge

There is a greater need to focus on the unique challenges male patients face with the COVID-19 pandemic (64). A recent study showed that there was a 29% reduction in men seeking medical treatment for ST-segment elevation myocardial infarction during the COVID-19 pandemic; in contrast, there was no change in women hospitalized (64). It is believed that the association of cardiovascular illness among men with COVID-19 may cause male patients to be reluctant in seeking medical attention for cardiovascular illness (64). Furthermore, the co-existence of COVID-19 symptoms may mask the symptoms of a heart attack among male patients (64). Male patients are also known to have a higher treatment-seeking threshold due to the prevalence of male stoicism and self-reliance (64). Therefore, physicians may be required to investigate other medical conditions in addition to COVID-19 to avoid under-diagnosing potential devastating medical conditions, such as heart disease, among male COVID-19 patients.

The current epidemiological data suggests that men are more affected than women by the COVID-19 virus (65). However, these studies are limited by location, sample size, and other potential biases in the population examined. Different clinical studies have given conflicting reports on the male or female predominance of COVID-19 infections. This discrepancy is likely due to the lack of large-scale epidemiological studies, socioeconomic disparities, or other confounders on the prevalence of pre-existing conditions in different countries. Recent epidemiological data from 38 countries showed a male predominance in COVID-19 infections, which increased in older age demographics. Furthermore, the case fatality rate was 1.7 times higher in men than females (65). The authors of the study suggested that differences in sex hormones, sex chromosomes, genetic polymorphisms, and epigenetic modifications between males and females may impact immune responses (65). Specifically, the authors reported that the ACE-2 receptor is located on the X chromosome and is down-regulated by increased estrogen levels (65). However, the expression of ACE-2 seems to increase with age and with the menstrual cycle of female mice. Therefore, it is possible that the increased expression of ACE-2 expression in elderly and pregnant patients may increase their risk for COVID-19. In general, the increased estrogen in females reduces their chance of viral infections from increase macrophage/neutrophil/dendritic cell activity, humoral response, and T-cell function compared to males. It is believed that the increased immune response in females reduces their susceptibility to COVID-19. The biological mechanism behind these differences also needs further elucidation.

## Conclusion

As the COVID-19 pandemic spreads, the differences between male and female mortality and infectivity remains an area of active investigation. The current literature suggests that men tend to have a higher risk of severe infection and mortality related to COVID-19. It is believed that elevated estrogen levels in female COVID-19 patients may reduce the severity and mortality of COVID-19 deaths through an elevation in the innate and humoral response. Furthermore, pre-clinical studies suggest that ACE-2 expression may increase the susceptibility of COVID-19 in pregnant patients. Similarly, the severity and mortality with COVID-19 differ between different ethnic groups. Although genetic polymorphisms associated with COVID-19 susceptibility among ethnic groups remains unknown, the increase in medical comorbidities and lack of access to care are significant contributors to increased COVID-19 mortality in these communities. As the pandemic continues to spread, African American and Hispanic communities have shown increased rates of infection and hospitalization compared to Caucasian populations. These differences are likely due to a higher prevalence of diabetes, hypertension, obesity, asthma, and heart disease in minority groups.

Further research is required to understand the molecular and pathophysiological mechanisms underlying these ethnic disparities in COVID-19 infection and severity. It remains unknown though whether genetic polymorphisms in ACE-2 expression or other genes may be involved in the gender or ethnic disparities to COVID-19 infection. Furthermore, the role of ACE-2 expression in male and female COVID-19 patients remains unknown. Pre-clinical studies show no difference in ACE-2 expression between male and female murine models. Additional studies are needed to evaluate whether expression levels of ACE-2 in the lung correlated with the severity or susceptibility of COVID-19 in human subjects. In the meantime, policies for reducing the spread of COVID-19 should take into consideration the unique challenges among men and women, including social, emotional, physical, and economic security.

## Author Contributions

AP and HG: conception and design. JK: first draft. All authors: literature review, critical revision and editing, and final approval.

## Conflict of Interest

The authors declare that the research was conducted in the absence of any commercial or financial relationships that could be construed as a potential conflict of interest.
